# Temporal characteristics of facial ensemble in individuals with autism spectrum disorder: examination from arousal and attentional allocation

**DOI:** 10.3389/fpsyt.2024.1328708

**Published:** 2024-02-19

**Authors:** Yuki Harada, Junji Ohyama, Misako Sano, Naomi Ishii, Keiko Maida, Megumi Wada, Makoto Wada

**Affiliations:** ^1^ Developmental Disorders Section, Department of Rehabilitation for Brain Functions, Research Institute of National Rehabilitation Center for Persons with Disabilities, Tokorozawa, Saitama, Japan; ^2^ Faculty of Humanities, Kyoto University of Advanced Science, Kyoto, Japan; ^3^ Human Augmentation Research Center, National Institute of Advanced Industrial Science and Technology, Kashiwa, Chiba, Japan; ^4^ Graduate School of Medicine, Nagoya University, Nagoya, Aichi, Japan; ^5^ Graduate School of Contemporary Psychology, Rikkyo University, Niiza, Saitama, Japan

**Keywords:** facial emotion, temporal ensemble, pupil size, eye tracking, eye-avoidance

## Abstract

**Introduction:**

Individuals with Autism Spectrum Disorder (ASD) show atypical recognition of facial emotions, which has been suggested to stem from arousal and attention allocation. Recent studies have focused on the ability to perceive an average expression from multiple spatially different expressions. This study investigated the effect of autistic traits on temporal ensemble, that is, the perception of the average expression from multiple changing expressions.

**Methods:**

We conducted a simplified temporal-ensemble task and analyzed behavioral responses, pupil size, and viewing times for eyes of a face. Participants with and without diagnosis of ASD viewed serial presentations of facial expressions that randomly switched between emotional and neutral. The temporal ratio of the emotional expressions was manipulated. The participants estimated the intensity of the facial emotions for the overall presentation.

**Results:**

We obtained three major results: (a) many participants with ASD were less susceptible to the ratio of anger expression for temporal ensembles, (b) they produced significantly greater pupil size for angry expressions (within-participants comparison) and smaller pupil size for sad expressions (between-groups comparison), and (c) pupil size and viewing time to eyes were not correlated with the temporal ensemble.

**Discussion:**

These results suggest atypical temporal integration of anger expression and arousal characteristics in individuals with ASD; however, the atypical integration is not fully explained by arousal or attentional allocation.

## 1 Introduction

An essential feature of individuals with autism spectrum disorder (ASD) is difficulty with social communication, which is related to an impaired ability to recognize facial emotions (1, 2). One method for investigating underlying mechanisms is the eye-tracking approach. Individuals with ASD tend to fixate on the eyes of a face for shorter periods than typically developing (TD) individuals (3, 4). This tendency has frequently been observed, especially in adults with ASD (5, 6). Given that attention towards the eyes enhances facial emotion recognition (7), atypical visual patterns would influence the impaired recognition of facial emotions.

Previous studies have proposed social salience and eye avoidance hypotheses to explain the atypical recognition of facial emotions in individuals with ASD. The social salience hypothesis presumes that individuals with ASD are less susceptible to socially informative areas (i.e. eyes; 8). Although the eyes provide viewers with mental states, intentions, and emotions (9), attention in individuals with ASD is not captured by eyes due to decreased social brain activity (10, 11). These individuals attend to physically salient parts of the face (e.g. the mouth), thus impairing recognition of facial emotions. In contrast, the eye-avoidance hypothesis presumes that social stimuli, especially for eyes with negative expressions, increase arousal levels in individuals with ASD (12). For instance, individuals with ASD are motivated to avoid eye regions to decrease arousal levels (4). This hypothesis is consistent with previous reports showing increased amygdala activity in individuals with ASD for fear expressions (13) and that autistic traits mainly impair the recognition of negative emotions (14). Stuart et al. (15) noted mixed evidence for these two hypotheses. Moriuchi et al. (16) conducted a cueing experiment, manipulating participants’ attention to focus on the eyes of facial images before presentation. They found that the time required to start eye avoidance did not differ between the ASD and TD groups. Additionally, they did not observe the eye avoidance when the participants’ attention was focused on the eyes of facial images by cueing method. Thus, it has been argued that the atypical recognition of facial emotions in individuals with ASD is related to arousal and attentional patterns, but the underlying mechanism remains unclear.

Recent studies investigated the characteristics of facial ensembles in individuals with ASD. Ensemble refers to perceiving an averaged item from multiple items arranged in different spatial or temporal positions (17). This ensemble perception has also been observed in facial emotions (18). Integrating multiple emotional expressions has various advantages for social communication. For example, to understand moods among members, it is necessary not only to recognize the facial emotions of each member but also to integrate them into a summary. Moreover, dynamic facial expressions should be integrally perceived to estimate others’ emotions because daily expressions change within a specific time window. Such integration is suggested to be difficult for individuals with ASD because of problems with global processing (19, 20) or bias to local processing (21). Therefore, autistic traits are considered to influence ensemble perception characteristics. Previous studies investigated the effects of autistic traits on spatial facial ensembles. Karaminis et al. (22) reported no differences in the facial ensembles between individuals with and without ASD. Recently, Chakrabarty and Wada (23) reported that half of the participants with the autism spectrum condition showed a decreased facial ensemble, while the other half did not. Although group differences in spatial ensembles are not apparent, there seem to be significant individual differences.

However, the effects of autistic traits on temporal facial ensembles remain unclear. Given the weak central coherence (19, 20) or local bias (21), individuals with ASD may perceive emotions based on a part of serial facial presentations rather than entire presentations. To investigate this issue, Harada et al. (24) examined whether autistic traits, measured using the Autism-Spectrum Quotient (AQ: 25, 26), influenced temporal facial ensembles. In this study, the participants viewed facial expressions serially switched between emotional and neutral. The participants evaluated the perceived intensity of the facial emotions for the overall presentation. They found that facial ensembles were influenced by the ratio of emotional expression time to total presentation time. Participants with higher autistic traits tended to score the perceived intensity of angry expressions as low (24). However, a higher AQ score does not always indicate a clinical diagnosis of ASD (27). This limitation should be resolved by clarifying group differences in the temporal ensemble of participants with and without a clinical diagnosis of ASD.

This study investigated the temporal ensemble characteristics of individuals with ASD. The experimental procedure was as described by Harada et al. (24). In this experiment, facial expressions were presented serially for three seconds and pseudo-randomly switched between emotional and neutral stimuli. The temporal ratio of the emotional expressions was manipulated as six levels. Subsequently, participants reported the intensity of their facial emotions. We also measured pupil size and attentional allocation during the presentation of facial images. Previous studies have asserted that the impaired recognition of facial emotions is related to atypical arousal and attentional patterns (8, 12). Therefore, we measured the perceived intensity of facial emotions (an indicator of the temporal ensemble), pupil size, and viewing time for facial images throughout the experiment. The pupil size is frequently used to measure arousal levels (28).

Three points must be verified: First, whether individuals with ASD would be less susceptible to the temporal ensembles of the perceived intensity of emotional expression than TD individuals. This is plausible because autistic traits impair the integration of multiple (or global) items (19, 20). Given that autistic traits impair the recognition of negative expressions (14), a lower susceptibility effect may be observed for negative emotions. Second, if the prediction was supported, we examined how the arousal level was related to the outcome. As pupil sizes reflect the arousal level (28), pupil sizes in individuals with ASD would be smaller for presenting facial expressions based on the social salience hypothesis but larger according to the eye-avoidance hypothesis. This is because the social salience hypothesis presumes that autistic traits decrease sensitivity to social information, whereas the eye-avoidance hypothesis presumes that these traits increase arousal to social information. Third, we examined whether viewing times for the eyes in facial images were shorter in individuals with ASD. Given these two hypotheses, individuals with ASD have shorter viewing times for eyes of the face.

## 2 Methods

### 2.1 Participants

Twenty individuals with ASD and 17 TD individuals (age and IQ were matched) participated in this study. The participants were naïve to the purpose of the study until they were debriefed. Before joining the research project, one of the authors, a graduate student, participated in the experiment. The participant was unaware of the study’s purpose. The sample size was determined based on previous studies (15–21 participants with ASD; 29–31). Written informed consent was obtained from all the participants. This study was approved by the Research Institute of the National Rehabilitation Center for Persons with Disabilities (approval number: 2020-012). Written informed consent was obtained from all participants.

The participants in the present study were broadly recruited from residents and university students near the institute. The participants were recruited under the following conditions; (1) age range of 15–40 years and (2) full-scale IQ of 80 or higher. Therefore, the participants completed the Japanese version (32) of the Wechsler Adult Intelligence Scale-III (33) For the TD group, participants were required not to have a diagnosis of a developmental disorders. For the ASD group, participants were required to have a diagnosis such as ASD, Asperger’s disorder, or Pervasive Developmental Disorder-Not Otherwise Specified, and the diagnosis was confirmed by the Japanese version (34) of the Autism Diagnostic Observation Schedule Component, Second Edition (ADOS-2; 35) as described below; An occupational therapist (M.S) with a research license for ADOS-2 assessed the ADOS-2 scores of each ASD participant in the ASD group.

The profile data of the participants with ASD are shown in **Table 1** (see **Table 2** for details of TD participants). Age and IQs were matched between the two groups [t (35) = 1.148, p = 0.259 for ages; t (35) = 1.318, p = 0.196 for verbal IQ; t (35) = 0.296, p = 0.769 for non-verbal IQ; t (35) = 0.647, p = 0.522 for full IQ]. Their ages ranged from 17 to 40 years old (M = 25.45, SD = 6.13) in participants with ASD and from 16 to 37 years old in TD participants (M = 23.29, SD = 5.12). The mean IQ in the participants with ASD was 110.70 (SD = 18.21) for the verbal, 106.00 (SD = 17.98) for non-verbal, and 110.30 (SD = 14.26) for full. The mean IQ in the TD participants was 117.65 (SD = 12.84) for the verbal, 104.53 (SD = 10.62) for non-verbal, and 113.12 (SD = 11.84) for full. The mean AQ was significantly higher in participants with ASD (M = 32.40, SD = 7.79) than in TD participants (M = 17.71, SD = 6.57) [t (35) = 6.136, p < 0.001]. All TD participants had AQ scores of < 30. The laterality quotient (LQ) for handedness (36) was matched between the two groups [t (35) = 0.054, p = 0.957]. Gender ratios were not significantly different between the ASD and TD groups (χ^2 ^= 0.079, p = 0.506, by a chi-square test). Therefore, we believe that age and gender variations have a restricted effect because these differences were not significant between the two groups, even though the present study includes a wide ranges of participant ages.

**Table 1:** Details of the 20 participants with ASD. All participants were residents of Japan (East Asian).

ID	Sex	Age	LQ	AQ	IQ			ADOS-2			Diagnosis	Medication
					Full	Verbal	Non-verbal	Comm	SI	Comm+SI		
#1	M	28	100	42	108	116	95	4	7	11	ASD	–
#2	M	17	100	21	110	86	140	3	7	10	ASD, ADHD, LD	–
#3	M	22	100	36	119	115	120	3	8	11	ASD	–
#4	M	18	53	36	103	110	94	2	8	10	ASD, ADHD	Atomoxetine, Clotiazepam, Suvorexant
#5	M	22	78	23	108	110	103	2	8	10	ASD	–
#6	F	40	100	34	100	104	95	3	6	9	ASD, ADHD, Anxiety disorder	Duloxetine, Diltiazem
#7	M	18	100	28	87	82	81	2	5	7	ASD, ADHD, Anxiety disorder, LD, Depression	Escitalopram, Aripiprazole
#8	M	24	100	42	99	109	87	3	4	7	ASD	–
#9	M	38	80	42	122	80	144	3	7	10	ASD, ADHD	Methylphenidate
#10	M	29	100	38	138	144	125	5	8	13	ASD	–
#11	M	27	100	25	122	134	103	4	6	10	ASD	–
#12	M	23	80	36	96	95	98	2	5	7	ASD	Atomoxetine
#13	F	28	89	31	110	110	108	2	5	7	ASD, ADHD	Zolpidem
#14	M	30	-20	18	100	112	84	2	5	7	ASD	Aripiprazole
#15	M	21	100	38	120	130	102	3	6	9	ASD	–
#16	M	25	79	21	90	95	91	6	5	11	ASD	–
#17	M	30	100	31	102	102	102	5	7	12	ASD, ADHD	Methylphenidate, Atomoxetine, Mosapride
#18	M	25	-100	35	110	110	108	2	7	9	ASD, Adjustment disorder	Brotizolam, Quetiapine, Amitriptyline, Fluoxetine
#19	F	19	79	43	122	130	106	4	8	12	ASD, Bipolar disorder, Tourette syndrome	Sodium Valproate, Guanfacine, Quetiapine
#20	F	25	89	28	140	140	134	2	5	7	ASD, ADHD	Dydrogesterone, Sulpiride

The symbol “-” in Medication column indicates no neuropsychiatric medication.

**Table 2:** Details of the 17 participants with TD.

ID	Sex	Age	LQ	AQ	IQ			Diagnosis	Medication
					Full	Verbal	Non-verbal		
#21	M	23	88	29	132	131	128		
#22	M	19	100	24	91	88	97		
#23	M	15	80	9	94	100	87		
#24	F	29	-40	10	121	121	116		
#25	M	24	100	16	122	129	108	Adjustment disorder Depressive disorder	
#26	M	19	79	18	116	120	106		
#27	M	22	89	15	101	111	88		
#28	M	19	76	23	118	131	98		
#29	F	22	100	22	102	107	95		
#30	M	26	68	11	108	112	101		
#31	M	21	80	29	118	123	106		
#32	M	22	-44	17	126	130	115		Levocetirizine
#33	F	29	100	6	108	111	103		
#34	M	21	89	14	122	129	108		
#35	F	21	100	20	124	134	108		
#36	F	28	100	19	102	106	97	Adjustment disorder	Paroxetine, bromazepam
#37	F	37	100	19	118	117	116		

The participant #32 have took Levocetirizine due to an anti-allergic medication.

All participants were residents of Japan (East Asian). Two participants had a diagnosis of adjustment disorder, but all did not have any diagnosis of developmental disorders. The Japanese version of ADOS-2 was not conducted for the TD participants.

### 2.2 Apparatus and stimuli

The apparatus and stimuli were the same as those described in Harada et al. (24). An LED monitor and desktop PC were used to conduct the study. The stimuli were presented on the monitor and controlled using MATLAB (MathWorks) with PsychToolbox (37–39). Eyelink 1000 PLUS (SR Research) was used to measure the participants’ pupil sizes and gaze positions.

Facial images of six basic emotions (anger, disgust, fear, happiness, sadness, and surprise) and neutral expressions were selected from the Facial Database of Advanced Industrial Science and Technology (40). A total of 42 facial images [(six emotions and neutral expressions) × six actors] were used.

The Japanese version of the AQ (26) was used to evaluate autistic traits. Participants were asked to indicate their agreement levels with 50 questions that described social communication scenes. Scoring was based on Wakabayashi et al. (26): the “agree” and “partial agree” responses to autistic items and “disagree” and “partial disagree” responses to inverted autistic items were counted.

### 2.3 Procedure

After instructions and informed consents, the participants sat on a chair in front of the monitor. The head was stabilised using the chin rest position. The participants performed a facial-emotion recognition task and a temporal ensemble task for approximately 60–80 minutes (including the resting time).

A facial emotion recognition task was conducted to confirm the recognition accuracy of static facial emotions. After the space key was pushed, a black fixation cross (‘+’) was presented at the centre of the monitor for one second. Subsequently, a facial image of any emotion and six choices (anger, disgust, fear, happiness, sadness, and surprise) were presented until responses. After the participants selected the most appropriate choice, the subsequent trial began. There were 72 trials (six facial emotion × six actors × two repetitions). In this task, the pupil size and gaze position were not recorded.

A nine-point calibration of eye tracking was conducted before the ensemble task. Participants were asked to fixate on each dot presented at any of nine locations (upper left, upper centre, upper right, middle left, middle centre, middle right, lower left, lower centre, and lower right of the monitor). If a corneal reflex image was not stably detected or the calculated gaze locations were largely biased with the actual location, the nine-point calibration was repeated up to three times. If the three trials failed, we conducted the ensemble task without eye tracking (six participants in the ASD group and three in the TD group). The number of participants without eye tracking was comparable to that of a previous study (24). Failures were most likely caused by equipping glasses to correct astigmatism.

**Figure 1:**
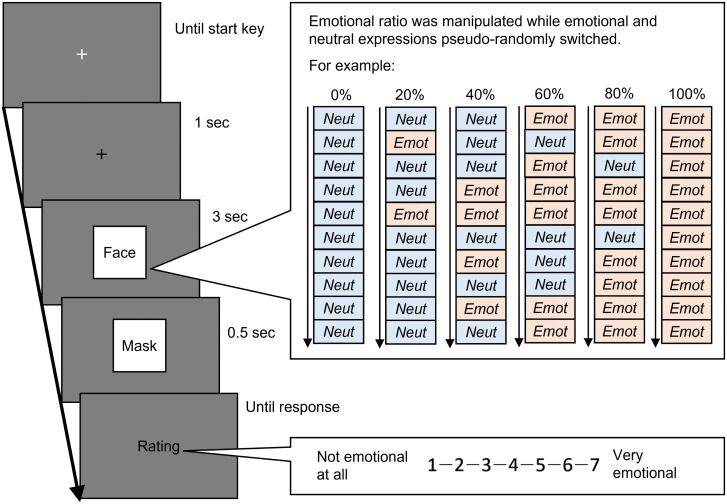
The trial sequence of the temporal ensemble task. In this task, the facial presentation comprised 10 frames that showed a neutral or one emotional expression. Facial expressions pseudo-randomly switched between neutral and emotion. The ratio of total duration for emotional expressions was manipulated as six levels. The inter-stimulus interval was 0. Ratios 0% and 100% indicate static neutral and emotional expressions, respectively.

The temporal ensemble task was performed to investigate the effect of autistic traits on the temporal facial ensemble. The trial sequence is shown in **Figure 1**. A black fixation cross (‘+’) was presented at the centre of the monitor. Subsequently, facial images of the same actor (7°× 7°of visual angle) were presented serially for three seconds. The facial presentation comprised 10 frames (0.3 seconds each), and the facial images were pseudo-randomly switched between emotional and neutral. The temporal ratio of emotional expressions to the total presentations was manipulated at six levels (0, 20, 40, 60, 80, and 100%). While viewing the facial images, the participants’ pupil size and gaze position were recorded (the sampling rate was set at 60 Hz by the PsychToolBox). After masking the stimuli, the participants estimated the perceived intensity of the facial emotions for the overall presentations using a seven-point scale (1 = not emotional to 7 = very emotional). The subsequent trial was initiated after a response. A total of 216 trials were conducted (six facial emotions × six ratios × six actors). We manipulated facial emotions between the experimental blocks and the ratio within each block. Although the order of facial emotions was not counterbalanced across participants, it did not differ between the ASD and TD groups significantly [χ2 (5) = 1.802, p = 0.876].

### 2.4 Analysis

The data obtained from the nine TD participants overlapped with those reported by Harada et al. (24). We analysed three dependent variables to examine our hypotheses: the perceived intensity of facial emotion, pupil size, and viewing time. The perceived intensity of the facial emotions was calculated by rating the data for the ensemble task. Pupil size is mainly composed of an initial reflex primarily influenced by the luminosity of the visual images and a later component (approximately 2 s after the stimulus onset: 28). The latter can be influenced by the arousal changes (41). Following previous studies, the average pupil size from 2 to 3 s after facial onset was analysed to evaluate the effect of emotional expressions on arousal. Data captured from 20–80% ratios were excluded from the analyses to control for the initial reflex. The pupil values captured from the eye tracker were converted into two types of data: the Z-score, which was calculated within the experimental block, and the relative data, which was derived from the pupil value (the value in 100% condition was divided by the mean values in 0% condition). The Z-score was employed to facilitate within-participants comparison, whereas the relative pupil size was utilised for between-group comparison. In the viewing-time analyses, two rectangles (120 pixel in width and 100 pixel in height) and a rectangle (280 pixel in with and 200 pixel in height) were set on eyes and a mouth as the areas of interest, respectively. For facial presentation, the gaze duration maintained in these areas was calculated as the viewing time of the eyes and mouth.

Statistical significance tests were conducted using software R (version 4.2.1). Analyses of Variance (ANOVAs) were conducted using the R function (anovakun version 4.8.9: 42). For multiple comparisons, the p-values were corrected using Holm’s method.

Before the analyses, we excluded eye data from 11 participants for the following reasons: the calibration failure in eye tracking (six participants with ASD and three TD participants) and corneal reflex detection in less than half of the total participants in some facial emotion conditions (two participants with ASD) mainly due to equipping glasses to correct astigmatism. Thus, we analysed the eye data from 12 participants in the ASD group and 14 in the TD group. To mitigate the effect of eye blinks, pupil data captured within ± 100 ms of a pupil miss were linearly interpolated, following the method described by Bradley et al. (28). Additionally, we excluded trials in which the interpolated data accounted for more than half of the total data points.

## 3 Results

For a comprehensive summary of the ANOVAs, please refer to **Table 3**.

**Table 3:** Summary of the Results of Analyses of Variance.

Figure	Independent variable	Factor	*F*	*p*	*ηp* ^2^
**Figure 2**	Performance of facial emotion recognition	Developmental group	6.161	.018*	.150
		Facial emotion	72.075	<.001*	.673
		Developmental group × Facial emotion	0.840	.523	.023
**Figure 3A**	Perceived intensity of emotion	Developmental group	0.047	.830	.001
		Ratio	47.084	<.001*	.574
		Facial emotion	11.407	<.001*	.246
		Developmental group × Ratio	0.175	.913	.005
		Developmental group × Facial emotion	0.900	.472	.025
		Facial emotion × Ratio	2.281	.004*	.061
		Developmental group × Facial emotion × Ratio	1.196	0.271	.033
**Figure 3B**	Ratio effect on the temporal ensemble	Developmental group	0.051	.823	.001
		Facial emotion	3.219	.008*	.084
		Developmental group × Facial emotion	1.982	.084	.054
**Figure 4**	Pupil size (Z-score) in participants with ASD	Facial emotion	0.577	.463	.050
		Ratio (100%: emotional vs. 0%: neutral)	0.128	.986	.012
		Facial emotion × Ratio	2.929	.021*	.210
**Figure 4**	Pupil size (Z-score) in TD participants	Facial emotion	0.008	.930	.001
		Ratio (100%: emotional vs. 0%: neutral)	1.069	.386	.076
		Facial emotion × Ratio	2.182	.067	.144
**Figure 4**	Pupil size (100% condition/0% condition)	Developmental group	0.328	.572	.014
		Facial emotion	2.817	.019*	.105
		Developmental group × Facial emotion	2.956	.015*	.110
**Figure 5B**	Viewing time rate for eyes	Developmental group	2.417	.133	.092
		Ratio	4.948	<.001*	.171
		Facial emotion	0.907	.479	.036
		Developmental group × Ratio	0.722	.608	.029
		Developmental group × Facial emotion	1.932	.094	.075
		Facial emotion × Ratio	1.147	.284	.046
		Developmental group × Facial emotion × Ratio	1.515	.053	.059
**Figure 5C**	Viewing time rate for a mouth	Developmental group	0.026	.874	.001
		Ratio	1.958	.090	.075
		Facial emotion	2.698	.024*	.101
		Developmental group × Ratio	2.719	.023*	.102
		Developmental group × Facial emotion	0.403	.846	.017
		Facial emotion × Ratio	0.799	.746	.032
		Developmental group × Facial emotion × Ratio	1.124	.309	.045

### 3.1 Pretest: Emotion recognition of static facial images

**Figure 2:**
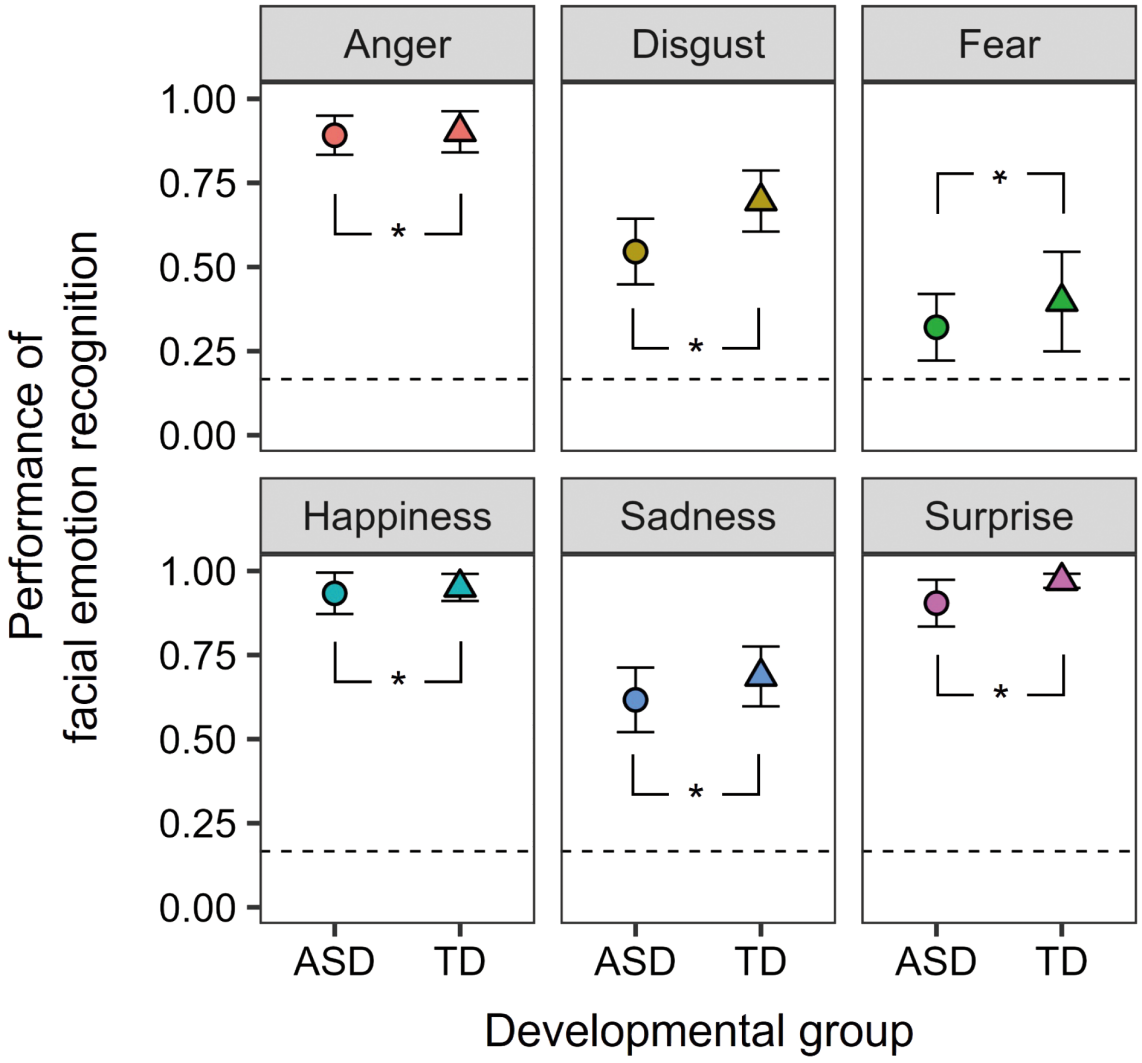
The mean facial emotion recognition task performance for the ASD and TD groups. The error bars represent 95% confidence intervals. Dashed lines represent the chance levels. Asterisks (*) represent significant differences between the two groups (p <.05).

**Figure 2** displays the performance of facial emotion recognition. This observation indicates that participants in both groups demonstrated a higher level of accuracy in recognizing facial emotions compared to the chance level. To confirm the effects of autistic traits, a two-way mixed-design ANOVA was conducted with the factors of developmental group (ASD and TD) and facial emotions (anger, disgust, fear, happiness, sadness, and surprise). The main effects of developmental group [F (1, 35) = 6.161, p = 0.0180, ηp^2 ^= 0.150] and facial emotions [F (5, 175) = 72.075, p < 0.0001, ηp^2 ^= 0.673] were significant. The former results show that individuals with ASD have difficulty recognizing facial emotions, irrespective of the emotion type. The two-way interaction was not significant [F (5, 175) = 0.840, p = 0.523, ηp^2 ^= 0.023]. Moreover, error patterns were analyzed in the Supplemental Information (**Supplementary Figure S1**).

### 3.2 Main test: temporal ensemble characteristics

#### 3.2.1 Perceived intensity of facial emotion

**Figure 3:**
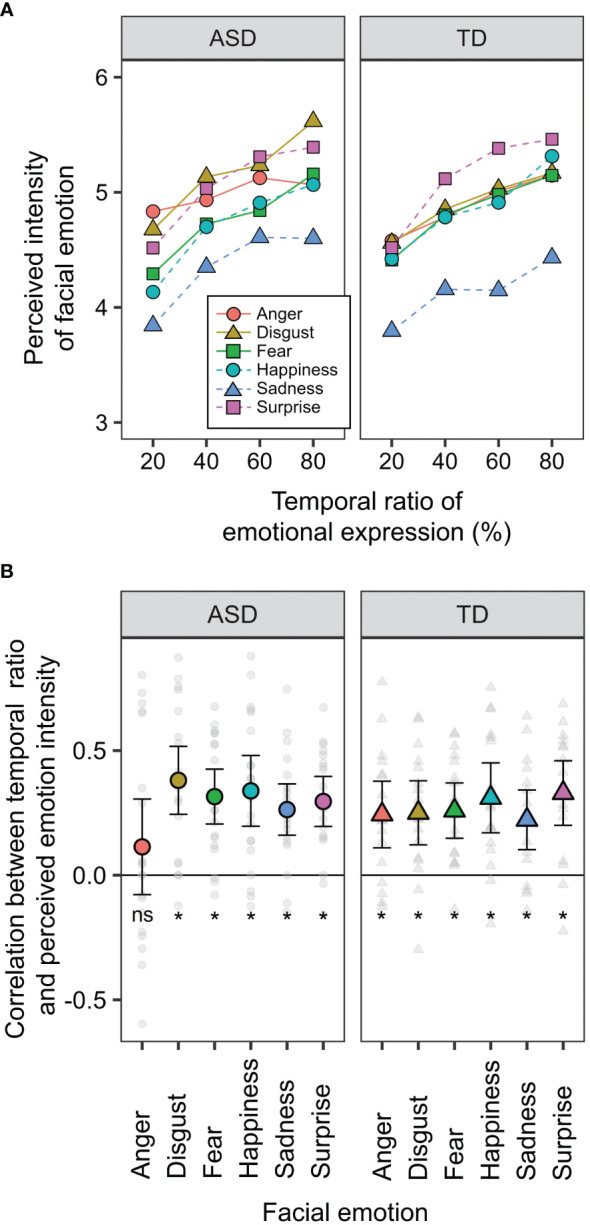
Behavioural data of the temporal ensemble task. **(A)** The means of perceived intensity of facial emotion as a function of the ratio of emotional expression. **(B)** The means of the ratio effect on the facial ensemble. The values were calculated as correlation coefficients between ratio and perceived intensity. Small dots indicate the data obtained from each participant. Error bars represent 95% confidence intervals. Asterisks (*) represent the values were significantly different from zero (p <.05), while "ns" represents there was no significant difference.

**Figure 3A** shows the perceived intensity of the facial emotions in the two groups. Ratios of 0 and 100% were excluded from the analysis because these conditions were static (complete data are shown in **Supplementary Figure S2**). A three-way mixed-design ANOVA was performed with the factors of developmental group, facial emotion, and ratio (20, 40, 60, and 80%). The main effects of facial emotion [F (5, 175) = 11.407, p < 0.0001, ηp^2 ^= 0.246] and ratio [F (3, 105) = 47.084, p < 0.0001, ηp^2 ^= 0.574] were significant. Furthermore, the two-way interaction between facial emotion × ratio was significant [F (15, 525) = 2.281, p = 0.004, ηp^2 ^= 0.061]. The other main effects and interactions were not significant (Fs < 1.195, ps > 0.271, ηp^2^s < 0.033). The perceived intensity of anger has been reported to correlate with the AQ score obtained from TD individuals (24). To confirm reproducibility, we calculated the correlation coefficients between the AQ score and perceived intensity in TD participants (**Supplementary Table S1**). The correlation coefficient for the anger condition was -0.337 [t (15) = 1.388, p = 0.185], which is comparable to the value (r = -0.353) obtained in the previous study. Although the present r-value was not significant, this could be because our sample size was smaller than that of the previous studies.

#### 3.2.2 Temporal ensemble

To examine the temporal ensemble characteristics in individuals with ASD, we calculated the correlation coefficients between the ratio and perceived intensity of facial emotions (**Figure 3B**). This correlation indicated the extent to which the temporal ensemble depended on the presentation time of the emotional expression (temporal ratio effect). If individuals with ASD perceived emotions based on a part of serial facial presentations rather than an entire presentation, the temporal ratio effect would decrease. T-tests were conducted between the values and zero (i.e. no correlation) to examine the temporal ratio effect. Multiplicity was corrected using Holm’s method. The results revealed that these values were significantly larger than zero (|t|s > 3.865, ps < 0.005), except for the anger condition in the ASD group [t (18) = 1.242, p = 0.228]. These results suggest that temporal ensembles in many individuals with ASD are less susceptible to the presentation times of angry expressions. This finding is consistent with our hypotheses. This may be related to atypical social perceptions of negative emotional faces among individuals with ASD. Tanaka and Sung (12) discussed that individuals with ASD are sensitive to angry expressions, which would increase arousal levels and cause them to avoid the eyes. The effects of arousal and attentional allocation on temporal ensembles are analyzed later to examine this possibility.

Moreover, a two-way mixed-design ANOVA was performed with the factors of developmental group and facial emotions. The main effect of facial emotion was significant [F (5, 175) = 3.219, p = 0.008, ηp^2 ^= 0.084], but that of the developmental group was not significant [F (1, 35) = 0.051, p = 0.823, ηp^2 ^= 0.001]. The two-way interaction was marginally significant [F (5, 175) = 1.982, p = 0.084, ηp^2 ^= 0.054]. The reason for this marginal significance may be that the five participants with ASD showed a more significant temporal ratio effect for angry expressions (**Figure 3B**). Individuals with ASD show more considerable individual differences in facial ensembles (23), which is consistent with the present results.

These results are partially inconsistent with those reported by Harada et al. (24). In the TD group, the temporal ratio effect was not significantly different between anger and other emotions; however, Harada et al. reported significant differences. A potential reason for this inconsistency is that we recruited TD participants based on AQ scores (AQ < 30), whereas the previous study included TD participants irrespective of their AQ scores. Given that autistic traits are related to the temporal ratio effect, the results of Harada et al. (24) may have been derived from participants with higher AQ scores.

#### 3.2.3 Arousal

**Figure 4:**
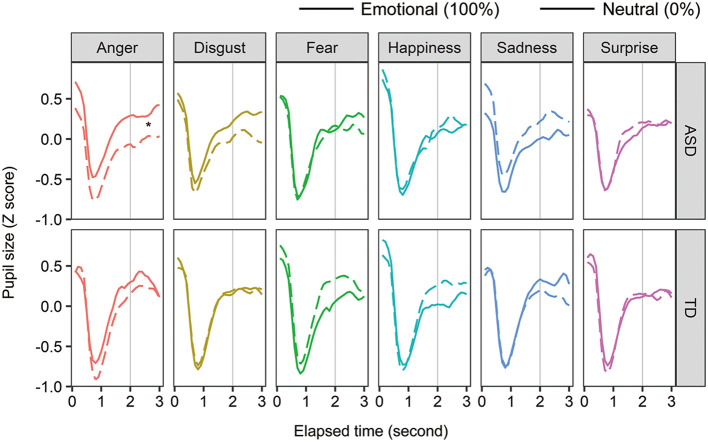
The mean change in pupil sizes for facial expressions. Asterisks (*) indicate significant differences (p <.05).

To examine the effect of facial emotions on arousal in individuals with ASD, pupil size was analyzed in two ways: within-participant and between-participant comparisons. For the within-group comparison, we calculated the Z-score of the pupil value in each facial emotion condition (**Figure 4**). We averaged the score from 2 to 3 s after facial expression onset. For each group, a two-way within-participant ANOVA was conducted with the factors of facial emotion and ratio (emotion:100%, neutral:0%). In the ASD group, the main effects of facial emotion and ratio were not significant (Fs < 0.577, ps > 0.463, ηp^2^ < 0.012), and the two-way interaction was significant [F (5, 55) = 2.929, p = 0.021, ηp^2 ^= 0.210]. For the anger condition, the simple main effect of ratio was significant [F (1, 11) = 8.434, p = 0.014, ηp^2 ^= 0.434] and pupil size in the ASD group was significantly larger for angry expression than for neutral expression. Although the simple main effect of facial emotion on emotional condition (100%) was significant [F (5, 55) = 2.396, p = 0.049, ηp^2 ^= 0.179], a multiple comparison test showed non-significant differences between facial emotions. Other simple main effects were not significant. For the TD group, neither any main effect nor the two-way interaction was significant (Fs < 2.182, ps > 0.067, ηp^2^s < 0.144). These within-participant comparisons show that individuals with ASD produce larger pupil sizes for angry expressions than neutral expressions, whereas typically developing individuals do not.

For between-participant comparison, we divided the pupil value in the 100% condition by the mean pupil value in the 0% condition. After facial expression onset, we averaged the relative pupil size from 2 to 3 s. A two-way mixed-design ANOVA was conducted with the factors of developmental group and facial emotion. The main effect of facial emotion and two-way interaction were significant [F (5, 120) = 2.817, p = 0.019, ηp^2 ^= 0.105; F (5, 120) = 2.955, p = 0.015, ηp^2 ^= 0.110], and the main effect of developmental group was not [F (1, 24) = 0.328, p = 0.572, ηp^2 ^= 0.014]. For sad expressions, the simple main effect of developmental group was significant [F (1, 24) = 4.327, p = 0.048, ηp^2 ^= 0.153]. This indicates that sad expression decreases arousal levels in individuals with ASD. For the ASD group, the simple main effect of facial emotion was significant [F (5, 55) = 3.507, p = 0.008, ηp^2 ^= 0.242], but a multiple comparison test showed no significant differences between facial emotions. Other simple main effects were not significant.

Participants with ASD showed increased arousal for angry expressions and decreased arousal for sad expressions. One possible explanation is that individuals with ASD are sensitive to arousal from facial stimuli. As angry expressions are more arousing (40) than other expressions, arousal in individuals with ASD would increase. Similarly, because sad expressions are less arousing, their arousal would decrease with presentation.

#### 3.2.4 Attentional allocation

**Figure 5:**
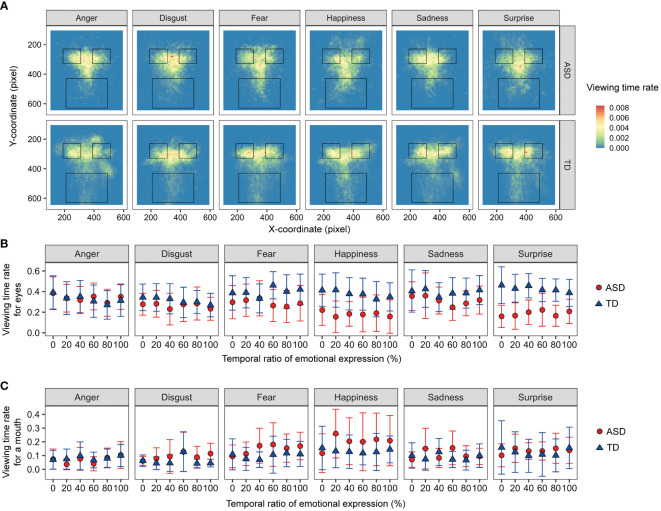
The viewing-time data for facial images. **(A)** Heat maps of viewing times. The three frames of black quadrangles indicate areas of interest for the eyes and mouth. **(B)** The means of viewing-time rates on the eye areas. **(C)** The means of viewing-time rates on the mouth area. Error bars represent 95% confidence intervals.

Viewing-time rates were analyzed to investigate the effects of autistic traits on attentional allocation. **Figure 5** shows the mean viewing time rates for the facial images. For the viewing time rates for eyes and mouth, a three-way mixed-design ANOVA was performed with the factors of developmental group, facial emotion, and ratio (0, 20, 40, 60, 80, and 100%). For viewing times on the eyes, the main effect of ratio was significant [F (5, 120) = 4.948, p < 0.001, ηp^2 ^= 0.171]. The other main effects and interactions were not significant (Fs < 1.932, ps > 0.053, ηp^2^s < 0.092). For the viewing times on a mouth, the main effect of facial emotion [F (5, 120) = 2.698, p = 0.024, ηp^2 ^= 0.101] and two-way interaction between developmental group and ratio [F (5, 120) = 2.719, p = 0.023, ηp^2 ^= 0.102] were significant. For the ASD group, the simple main effect of ratio was significant [F (5, 55) = 3.225, p = 0.0127, ηp^2 ^= 0.227], but a multiple comparison test showed non-significant differences between ratios. The other main effects and interactions were not significant (Fs < 1.958, ps > 0.090, ηp^2^s < 0.075). The results showed no difference in attentional allocation between individuals with and without ASD.

#### 3.2.5 Relationship between arousal and temporal ensemble

**Figure 6:**
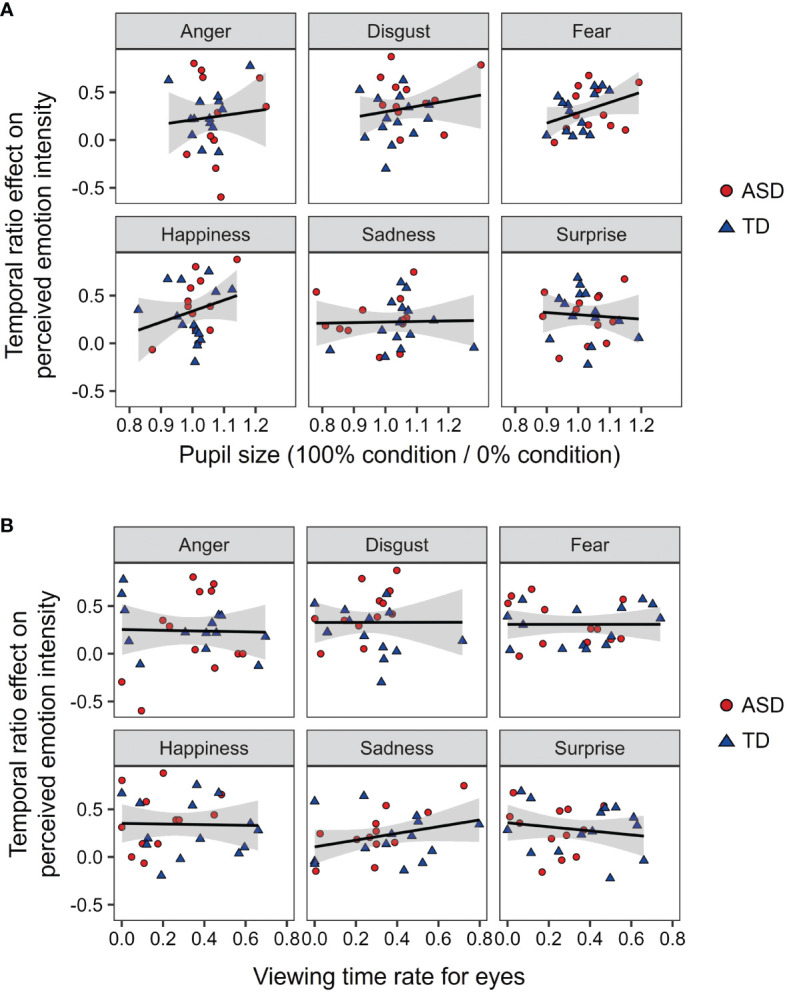
The relationship between the temporal ensemble, arousal, and attentional allocation. **(A)** The relationship between the pupil size and temporal ratio effect. **(B)** The relationship between the viewing times for eyes and temporal ratio effect. An asterisk (*) represents the significant correlation (p <.05).

To discuss the mechanisms underlying the effect of autistic traits on temporal ensembles, we examined the effects of pupil size and viewing time for eyes on the temporal ensemble of facial emotions (**Figure 6**). For each facial emotion, we conducted a multiple regression analysis on the temporal ratio effect with developmental group, pupil size, and viewing times for the eyes as predictors. The results showed that none of the models were significant (Fs < 1.322, ps > 0.293, R^2^s < 0.153). This suggests that the rating of the temporal ensemble was not fully explained by arousal or attentional allocation.

## 4 Discussion

This study investigated the temporal ensemble characteristics of emotional expression in individuals with ASD. The temporal ensemble of many participants with ASD was less susceptible to the temporal ratio of angry expressions. This finding is consistent with our hypotheses. Moreover, **Figure 6A** shows that pupil size and viewing time did not affect the temporal ratio effect. These results suggest that anger weakens the temporal ensemble in individuals with ASD; however, arousal or attention to the eyes cannot fully explain this effect.

We found that individuals with ASD have difficulty averaging facial emotion of anger recognition. This finding could be used to assess autistic traits from the perspective of temporal facial ensembles. Recent studies have proposed behavioral assessments using computational models of facial expressions (43, 44). Leo et al. (44) developed a computational model that can evaluate individuals’ abilities to express their emotional states by analyzing the muscle conditions of facial parts according to computer vision and machine learning technologies. This idea can be applied to facial emotion recognition. For example, a typical ensemble of neutral-anger expressions can be modelled using data obtained from individuals without ASD. This model is useful for quantitatively assessing how atypical individuals’ temporal ensemble abilities are. Furthermore, the findings of this study may be useful for developing tools that support communication in situations such as online meetings. If the temporal ensemble of angry facial expressions causes excessive arousal, then tempering the display may facilitate communication, and vice versa.

The pupil data produced two results regarding whether individuals with ASD are sensitive to emotional expression. The participants with ASD showed a more significant size of pupils while viewing angry expressions but a smaller size while viewing sad expressions. As noted above, previous studies have proposed reported mixed evidence: Participants with ASD have a smaller pupil size (45) and a larger one (46) for facial expressions. Our results may integrate the mixed results into the idea that individuals with ASD are sensitive to stimuli arousal levels. Angry expressions are more arousing, whereas sad expressions are less arousing (40). Accordingly, a larger pupil size for angry expressions and a smaller one for sad expressions are considered sensitive responses, which might be related to hypersensitivity in individuals with ASD. This idea can explain some of the mixed results but does not explain other results (e.g., 11). However, further studies are required to examine this hypothesis.

Alternatively, we can interpret that smaller pupil size for sadness stems from decreased affective empathy in individuals with ASD. Empathy has been discussed in the context of both affective and cognitive empathy (47). The pupillary response is considered to be influenced by affective empathy because it is under the control of the automatic nervous system. Interestingly, social responsiveness has been reported to be related to sensitivity to sad expressions (48), demonstrating the effect of affective empathy on responses to sadness. However, we did not observe any specific effect of sadness on the facial emotion recognition (**Figure 2**) or the temporal ensemble (**Figure 3B**). This suggests that individuals with ASD can recognize sad expressions but may show atypical emotional responses.

Viewing times for eyes did not differ between individuals with ASD and those with TD, which is inconsistent with previous results. Studies using eye-tracking have shown that individuals with ASD fixate on the eyes of static facial images for shorter durations (49). One possible reason for these inconsistent results is that our stimuli may have caused dynamic perceptions. Unlike static facial expressions, dynamic facial expressions are less likely to cause shorter fixations on the eyes of facial images (50). Our facial images switched between emotional and neutral images, which may attract attention in individuals with ASD.

This study had three potential limitations. First, the present study includes wide ranges of participant ages, and we did not examine whether the results can be generalized to different age groups of individuals with ASD. As the participants with ASD were older than 16 years, the present results represent trends in adolescents and adults with ASD. It has been suggested that atypical recognition processes of facial emotions change with age. Black et al. (49) found that children with ASD less produced shorter fixations on the eyes of faces (51, 52), although adolescents and adults with ASD more produced them. Moreover, increased arousal may stem from negative social feedback (49). Such social effects increase during adolescence (e.g., social anxiety: 53). Future studies should include children with ASD to examine the impact of autistic traits on temporal ensembles. Second, the diversity of the participants with ASD may have influenced the results. Some participants with ASD had clinical symptoms (e.g. ADHD, anxiety disorders, and learning disorders) and were taking psychoactive drugs (e.g. methylphenidate). The co-occurrence of clinical symptoms is common in individuals with ASD (54). Therefore, it is difficult to fully attribute the present results to autistic traits. Third, a simplified procedure was used to measure the temporal ensembles. As Harada et al. (24) noted, the classical approach for a temporal ensemble presents facial images with a manipulated emotional intensity. In contrast, we presented emotional and neutral images to measure the temporal ratio effect. Future studies should use the classical temporal ensemble procedure to address this limitation.

## 5 Conclusion

The following two significant results were obtained: First, many individuals with ASD have temporal ensembles less susceptible to the temporal ratio of angry expressions. Second, individuals with ASD produced a greater size of pupils when viewing angry expressions and a smaller size of pupils when viewing sad expressions. However, pupil size and viewing times for eyes of faces did not predict the temporal ensembles. These results suggest that (a) autistic traits influence the temporal integration of angry expressions, (b) individuals with ASD show atypical emotional responses that are sensitive to facial expressions, and (c) the temporal ensemble of facial emotions is not fully explained by arousal or attentional allocation.

## Data availability statement

The datasets presented in this study can be found in online repositories. The names of the repository/repositories and accession number(s) can be found below: https://osf.io/hkcys/.

## Ethics statement

The studies involving humans were approved by Ethics Committee of the National Rehabilitation Center for Persons with Disabilities. The studies were conducted in accordance with the local legislation and institutional requirements. Written informed consent for participation in this study was provided by the participants’ legal guardians/next of kin.

## Author contributions

YH: Conceptualization, Data curation, Formal analysis, Funding acquisition, Investigation, Methodology, Software, Validation, Visualization, Writing – original draft. JO: Conceptualization, Funding acquisition, Methodology, Resources, Writing – review & editing. MS: Methodology, Writing – review & editing. NI: Methodology, Resources, Validation, Writing – review & editing. KM: Methodology, Writing – review & editing. MeW: Methodology, Writing – review & editing. MaW: Conceptualization, Data curation, Funding acquisition, Project administration, Supervision, Validation, Writing – review & editing.
