# Supplementary material for: Temporal characteristics of facial ensemble in individuals with autism spectrum disorder: examination from arousal and attentional allocation
Source: Front Psychiatry. 2024 Feb 19;15:1328708. doi: 10.3389/fpsyt.2024.1328708 (PMC10910007; doi:10.3389/fpsyt.2024.1328708)
Supplement: Supplementary file 1 [file DataSheet_1.docx]

Supplementary Material

Temporal Characteristics of Facial Ensemble in Individuals with Autism Spectrum Disorders: Examination from Arousal and Attentional Allocation

**Yuki HARADA^1,2^, Junji OHYAMA^3^, Misako SANO^4^, Naomi ISHII^1^, Keiko MAIDA^1^,**

**Megumi WADA^1,5^, and Makoto WADA^1^***

^1^ Developmental Disorders Section, Department of Rehabilitation for Brain Functions, Research Institute of National Rehabilitation Center for Persons with Disabilities, Tokorozawa, Saitama 359-8555, Japan

^2^ Faculty of Humanities, Kyoto University of Advanced Science, Kyoto city, Kyoto 615-0096, Japan

^3^ Human Augmentation Research Center, National Institute of Advanced Industrial Science and Technology, Kashiwa, Chiba 277-0882, Japan

^4^ Graduate School of Medicine, Nagoya University, Nagoya, Aichi 461‑8673, Japan

^5^ Graduate School of Contemporary Psychology, Rikkyo University, Niiza, Saitama 352-0003, Japan

*** Correspondence:**

Makoto WADA (ORICD: 0000-0002-2183-5053)

Email: [wada-makoto@rehab.ge.jp](mailto:wada-makoto@rehab.ge.jp)

# Supplementary Tables

# Table S3. Correlation coefficients between the AQ score and perceived intensity of facial emotion.

| Developmental group | Emotion | *r* | *t* | *df* | *p* |
| --- | --- | --- | --- | --- | --- |
| Merged | Anger | -.048 | -0.283 | 35 | 0.779 |
| Merged | Disgust | .134 | 0.799 | 35 | 0.430 |
| Merged | Fear | -.114 | -0.679 | 35 | 0.502 |
| Merged | Happiness | -.149 | -0.891 | 35 | 0.379 |
| Merged | Sadness | .059 | 0.348 | 35 | 0.730 |
| Merged | Surprise | -.074 | -0.438 | 35 | 0.664 |
| ASD | Anger | .010 | 0.043 | 18 | 0.966 |
| ASD | Disgust | .140 | 0.602 | 18 | 0.555 |
| ASD | Fear | -.087 | -0.369 | 18 | 0.716 |
| ASD | Happiness | -.137 | -0.587 | 18 | 0.564 |
| ASD | Sadness | -.075 | -0.319 | 18 | 0.753 |
| ASD | Surprise | -.092 | -0.394 | 18 | 0.698 |
| TD | Anger | -.337 | -1.388 | 15 | 0.185 |
| TD | Disgust | -.115 | -0.448 | 15 | 0.661 |
| TD | Fear | -.150 | -0.586 | 15 | 0.566 |
| TD | Happiness | -.128 | -0.499 | 15 | 0.625 |
| TD | Sadness | -.050 | -0.192 | 15 | 0.850 |
| TD | Surprise | -.052 | -0.202 | 15 | 0.843 |

# Supplementary analyses

The error pattern to the pretest is shown in Fig S1. A linear-mixed model analysis was performed on the identification rates with fixed effects of developmental group (ASD, TD), facial emotion (anger, disgust, fear, happiness, sadness, surprise), and identified emotion (anger, disgust, fear, happiness, sadness, surprise). The final model showed that the main effect of identified emotion [F (5, 1260) = 55.987, p < .0001] and two-way interactions of developmental group × identified emotion [F (5, 1260) = 2.229, p = .0493] and facial emotion × identified emotion [F (25, 1260) = 359. 00, p < .0001] were significant. More importantly, the three-way interaction was also significant [F (25, 1260) = 1.693, p = .0180]. The multiple comparison showed that the disgust, fear, and sadness expressions were inaccurately recognized in the participants with ASD more significantly than in the TD participants [t (1260) = 4.335, p < .0001 for disgust; t (1260) = 2.199, p = .0280 for fear; t (1260) = 2.008, p = .0448 for sadness].


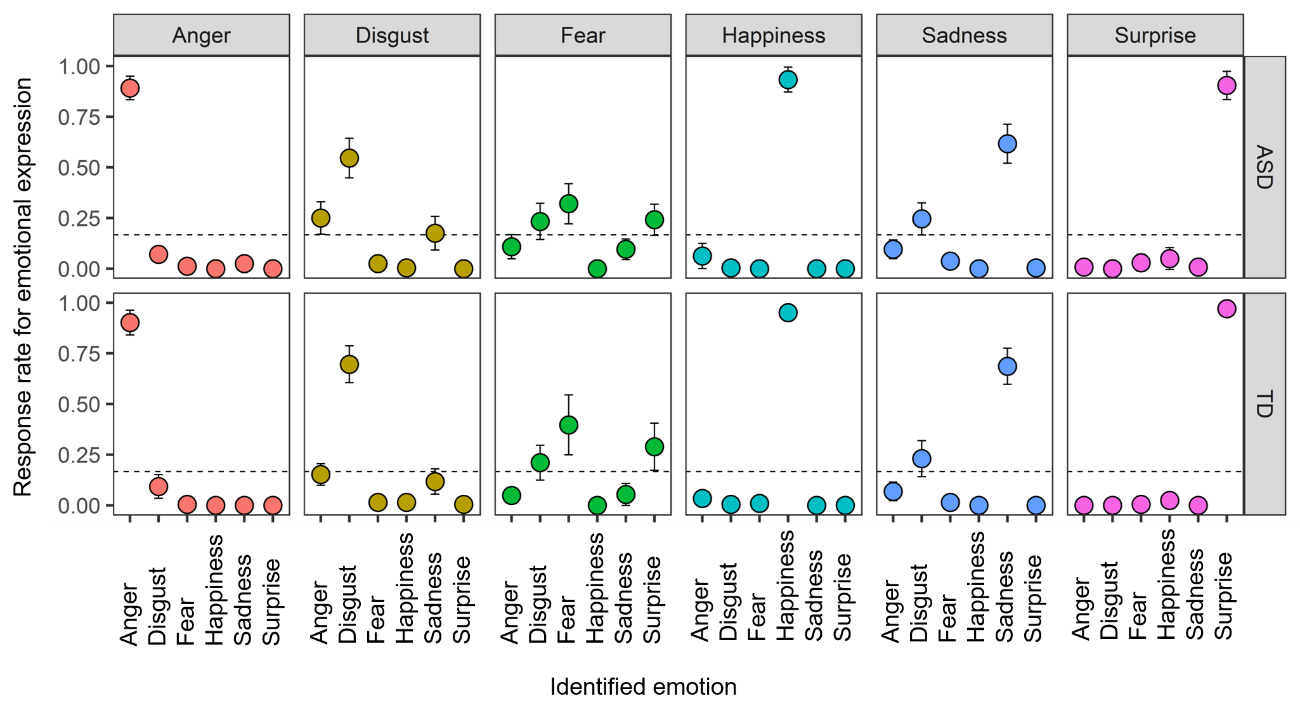


**Fig S1.** Response rates for emotional expressions in the pretest. Upper panel shows the data for participants with ASD, and the lower one shows those for TD participants. Horizontal panels show emotions of facial images. Horizontal axis shows the identified facial emotion. Error bars represent 95% confidence intervals.


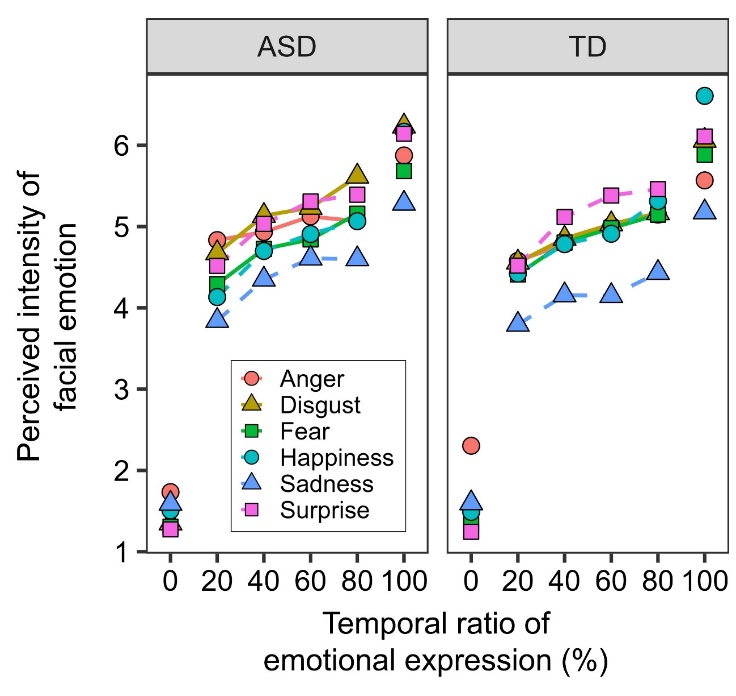


**Fig S2.** Perceived intensity of facial emotion as a function of temporal ratio of emotional expression.
